# Factors Affecting Volume Changes of the Somatosensory Cortex in Patients with Spinal Cord Injury: To Be Considered for Future Neuroprosthetic Design

**DOI:** 10.3389/fneur.2017.00662

**Published:** 2017-12-11

**Authors:** Yvonne Höller, Arijan Tadzic, Aljoscha C. Thomschewski, Peter Höller, Stefan Leis, Santino Ottavio Tomasi, Christoph Hofer, Arne Bathke, Raffaele Nardone, Eugen Trinka

**Affiliations:** ^1^Department of Neurology, Christian Doppler Medical Centre and Centre for Cognitive Neuroscience, Paracelsus Medical University Salzburg, Salzburg, Austria; ^2^Spinal Cord Injury and Tissue Regeneration Center, Paracelsus Medical University, Salzburg, Austria; ^3^Department of Neurosurgery, Paracelsus Medical University of Salzburg, Salzburg, Austria; ^4^Wavelab, Department of Computer Sciences, University of Salzburg, Salzburg, Austria; ^5^Department of Mathematics, University of Salzburg, Salzburg, Austria; ^6^Department of Neurology, Franz Tappeiner Hospital, Merano, Italy

**Keywords:** spinal cord injury, somatosensory cortex, volume, neuroplasticity, segmentation

## Abstract

Spinal cord injury (SCI) leads to severe chronic disability, but also to secondary adaptive changes upstream to the injury in the brain which are most likely induced due to the lack of afferent information. These neuroplastic changes are a potential target for innovative therapies such as neuroprostheses, e.g., by stimulation in order to evoke sensation or in order to suppress phantom limb pain. Diverging results on gray matter atrophy have been reported in patients with SCI. Detectability of atrophy seems to depend on the selection of the regions of interest, while whole-brain approaches are not sensitive enough. In this study, we discussed previous research approaches and analyzed differential atrophic changes in incomplete SCI using manual segmentation of the somatosensory cortex. Patients with incomplete SCI (ASIA C-D), with cervical (N = 5) and thoracic (N = 6) injury were included. Time since injury was ≤12 months in 7 patients, and 144, 152, 216, and 312 months in the other patients. Age at the injury was ≤26 years in 4 patients and ≥50 years in 7 patients. A sample of 12 healthy controls was included in the study. In contrast to all previous studies that used voxel-based morphometry, we performed manual segmentation of the somatosensory cortex in the postcentral gyrus from structural magnetic resonance images and normalized the calculated volumes against the sum of volumes of an automated whole-head segmentation. Volumes were smaller in patients than in controls (*p* = 0.011), and as a tendency, female patients had smaller volumes than male patients (*p* = 0.017, uncorrected). No effects of duration (subacute vs. chronic), level of lesion (cervical vs. thoracic), region (left vs. right S1), and age at onset (≤26 vs. ≥50 years) was found. Our results demonstrate volume loss of S1 in incomplete SCI and encourage further research with larger sample sizes on volumetric changes in the acute and chronic stage of SCI, in order to document the moderating effect of type and location of injury on neuroplastic changes. A better understanding of neuroplastic changes in the sensorimotor cortex after SCI and its interaction with sex is needed in order to develop efficient rehabilitative interventions and neuroprosthetic technologies.

## 1 Introduction

Spinal cord injury (SCI) induces a loss of both motor and sensory function below the level of injury (1). This leads to a degenerative development along the descending and ascending pathways transferring information between the spinal cord and the brain (2–7).

Spinal cord atrophy is the result of a combination of pathophysiological processes, including loss of neurons as well as axonal demyelination, degeneration, and dieback (8). Absent sensory inputs after spinal cord injuries lead to instantaneous and progressive functional, structural, and molecular changes in the central nervous system (9). At the early stage of the injury, the majority of the neurons remain intact, but there is no regeneration of axons (10, 11). Several studies reported atrophy of the cortex and the spinal cord in patients with SCI, additionally to a loss of axonal integrity (1–3, 5, 6, 8, 10–18).

In rats following SCI, Wall and Egger discovered in 1971 that discontinuation of cortical loops in rats following SCI gives rise to reorganization and formation of new connections of cortical structures (19). The ability for reorganization of the brain following impairment, such as SCI, is called neuroplasticity [reviewed in Ref. (20)]. However, cortical plasticity is not always helpful. First, the maladaptive neuroplasticity can lead to phantom sensations or pain (21, 22). Moreover, it can be expected that even if one day it would be possible to restore the lesioned pathways in the spinal cord, or to bypass them with neuroprosthetic devices, the neuroplastic changes in the sensorimotor cortex could prevent the patients from a fast recovery because the newly regenerated axons may project to brain areas that are remapped in order to serve other functions.

However, cortical reorganization alone may not prevent therapeutic strategies such as neuroprostheses to be implemented successfully. More so, loss of brain matter in the sense of progressive cortical atrophy could represent a serious problem, for example, when brain–computer interfaces or neuroprostheses are directly interfacing with the central nervous system (23, 24).

Loss of white and gray matter has been reported in patients with SCI in several studies (3, 5, 17, 18, 25–28). The studies reported cortical atrophy in diverging areas, including primary motor cortex, primary somatosensory cortex, and supplementary motor cortex. One study demonstrated that the volumetric change is detectable also in a longitudinal design in the acute and subacute stage of the first year (18). The earliest study in this field applied manual measurement of cortical thickness and voxel-based morphometry and found no differences between patients with SCI and healthy controls (29). The other studies all relied on voxel-based morphometry. All but the most recent one (30) of these studies reported reduced volume in sensorimotor areas, but the results varied in the exact location of these differences.

A source of this inconsistency might be the exact use of voxel-based morphometry. The technique may be applied to regions of interest or to the whole brain, with statistically significant clusters being submitted to further analysis of group differences. It seems that the approach used, either by globally searching for differences or by limiting the test to regions of interest, has a major effect on the detectability of differences. Most studies found significant differences within the sensorimotor areas by applying a region of interest analysis (5, 17, 18, 26–29). Most interestingly, two studies reported that with whole-brain analysis, no differences in the sensorimotor region were found, while analyzing only the regions of interest yielded the expected results (27, 28). Furthermore, the only study not implementing any region of interest analysis did not report any significant results in the sensorimotor areas (30).

The diverging results strongly suggest that the changes are small, and that atrophy in other brain regions may be more prominent; as a consequence, the small changes in the sensorimotor area might not reach significance on the global cortical or brain level. Thus, the choice of the region of interest might largely moderate the findings. However, a manual analysis of cortical thickness found no results even when restricted to the primary motor cortex (29).

A further explanation may be that the studies involved quite different samples. It seems plausible that the level of injury (cervical vs. lower injury), time since injury, type of injury (traumatic vs. non-traumatic), and completeness of the injury could play a role. However, Jutzeler et al. reported no difference in atrophy between patients with complete and incomplete SCI (28) using voxel-based morphometry. Nevertheless, gray matter volumes differ with respect to the presence of neuropathic pain (27, 28).

Despite several studies reported cortical atrophy in patients with SCI, the effect of level of injury, duration, age, and sex where only rarely addressed. It is unclear why manual measurement would not yield an effect, while automated analysis is able to document significant results. In a recent project, we could demonstrate that the type of automated segmentation as well as the underlying pathology may moderate the accuracy of the delineation of the region of interest (31, 32). Accordingly, atrophic regions may be identified with greater difficulty. Therefore, in the present study, we aimed to assess manual segmentations of volumes of the postcentral gyrus in patients with incomplete cervical vs. thoracic SCI, at a subacute vs. chronic stage, and to test for effects of age and sex.

## 2 Materials and Methods

### 2.1 Ethics

This study was carried out in accordance with the recommendations of Good Clinical Practice.

From participants who were involved during a scientific study, we obtained written informed consent in accordance with the Declaration of Helsinki. The protocol was approved by the Ethics Commission Salzburg (Ethikkommission Land Salzburg; approval number 1541).

From the other participants who were not involved in the scientific studies we used clinical routine data retrospectively, extracted from the clinical database in anonymized form.

### 2.2 Sample

We identified 21 patients with SCI having been between 2005 and 2015 at the Department of Neurology, Christian Doppler Medical Centre, Paracelsus Medical University for clinical routine examinations or having participated in the scientific study as mentioned in Section 2.1. From the clinical routine examinations, we identified all patients with T1-weighted MRI volumes recorded after the injury. From this sample, 4 patients had to be excluded because they suffered from other diseases affecting the central nervous system (1 MS, 1 hypoxic encephalopathy, 1 unresponsive wakefulness syndrome, and 1 with kryptogenic epilepsy). Another 5 patients had to be excluded because the MRI was done within 1 week after injury, so that we did not expect any atrophic changes in these patients. Another patient was excluded because of insufficient quality of the MRI. The clinical details of this final sample are listed in Table 1.

**Table 1:** Clinical details and MRI-sequence of the patients with SCI included in the study.

nr.	Age	Sex	Time	MRI	Etiology	AIS grade	Level
1	50	m	2	MPR	Cervical myopathy; laminectomy	D	C4
2	26	m	216	MPR	Car accident	C–D^a^	C6
3	22	m	312	MPR	Sports accident	C–D^a^	C6
4	69	m	12	MPR	Spinal ischemia TH8	D^b^	T9
					Arteriovenous fistula TH4–8		
					Laminectomy L4/L5		
5	22	f	144	FFE Sag	Fracture	D^b^	T11
					Motorcycle accident		
6	20	m	152	3D TFE ISO Sag	T-cell lymphoma	D^b^	T3
7	85	f	1	3D TFE ISO Sag	Unstable odontoid	D^b^	C4
					Fracture		
8	53	m	6	3D TFE ISO Sag	Spinal ischemia	D	T5
9	59	f	1	3D TFE ISO Sag	Spinal ischemia	D	T6
10	53	m	2	3D TFE ISO Sag	Fracture, luxation	D	C4
11	57	f	3	FFE Sag	Arteriovenous fistula	D	T10

*age, at onset; level, of injury; time, since injury/onset given in months; MRI, magnetic resonance imaging, T1-weighted image sequence; AIS Grade, according to ASIA—American Spinal Injury Association; level, neurological level (sensory symptoms below this level); MPR, multi-planar reconstructed; FFE, fast field echo; TFE, turbo field echo; Sag, saggital*.

*^a^No AIS grade determined; patients used a wheelchair and were unable to walk but could use their arms; preserved sensory function in arms and legs*.

*^b^No AIS grade was determined; rating was done post hoc based on detailed information from diagnostic documentation*.

In addition, we included MRIs of 12 healthy controls (age range 19–35 years, median 23 years; 7 female) who were recruited among the students of the Universities of Salzburg (Austria).

#### 2.2.1 MRI

T1-weighted MRI volumes were acquired at Siemens (Erlangen, Germany) Magnetom TrioTim syngo MR B17 at 3 Tesla for all healthy controls and patients 1–4 using a 12-channel head coil and the following parameters: sagittal orientation, 192 slices per slab, 256 mm FoV read at 93.8% phase, repetition time (TR) = 2,300 ms, echo time (TE) = 2.91 ms, inversion time (TI) = 900 ms, flip angle (FA) = 9 deg, and a slice thickness of 1 mm resulting in a voxel size of 1 × 1 × 1 mm^3^. One MRI—of patient 5—was acquired at a Philips (Hamburg, Germany) 1.5 T NT Intera: fast field echo sagittal, slice thickness = 4 mm, TR = 198.83 ms, TE = 2.50, imaging frequency = 63.90, number of phase encoding steps = 154, FA = 90 deg; The other MRIs were acquired at a Philips (Hamburg, Germany) 3 T Achieva: Patient 6,7: 3D turbo field echo ISO sagittal, slice thickness = 1 mm, TR = 8.09 ms, TE = 3.70 ms, imaging frequency = 127.76, number of phase encoding steps = 240, FA = 8 deg; Patient 8: 3D turbo field echo ISO sagittal, slice thickness = 1 mm, TR = 8.13 ms, TE = 3.73 ms, imaging frequency = 127.79, number of phase encoding steps = 240, FA = 8 deg; Patient 9: 3D turbo field echo ISO sagittal, slice thickness = 1 mm, TR = 8.29 ms, TE = 3.8 ms, imaging frequency = 127.78, number of phase encoding steps = 240, FA = 8 deg; Patient 10: 3D turbo field echo ISO sagittal, slice thickness = 1 mm, TR = 8.25 ms, TE = 3.8 ms, imaging frequency = 127.75, number of phase encoding steps = 240, FA = 8 deg; Patient 11: fast field echo sagittal, slice thickness = 5 mm, TR = 315 ms, TE = 4.60, imaging frequency = 127.77, number of phase encoding steps = 320, FA = 80 deg.

### 2.3 Manual Segmentation

The manual segmentation of the MRI volumes was performed on a Wacom Cintiq 22 inch HD interactive pen display (resolution 1,920 × 1,200) and using the 64-bit 3DSlicer software for Windows 7 (v. 4.2.2-1 r21513) in order to label the somatosensory cortex voxels for each slice independently.

**Figure 1:**
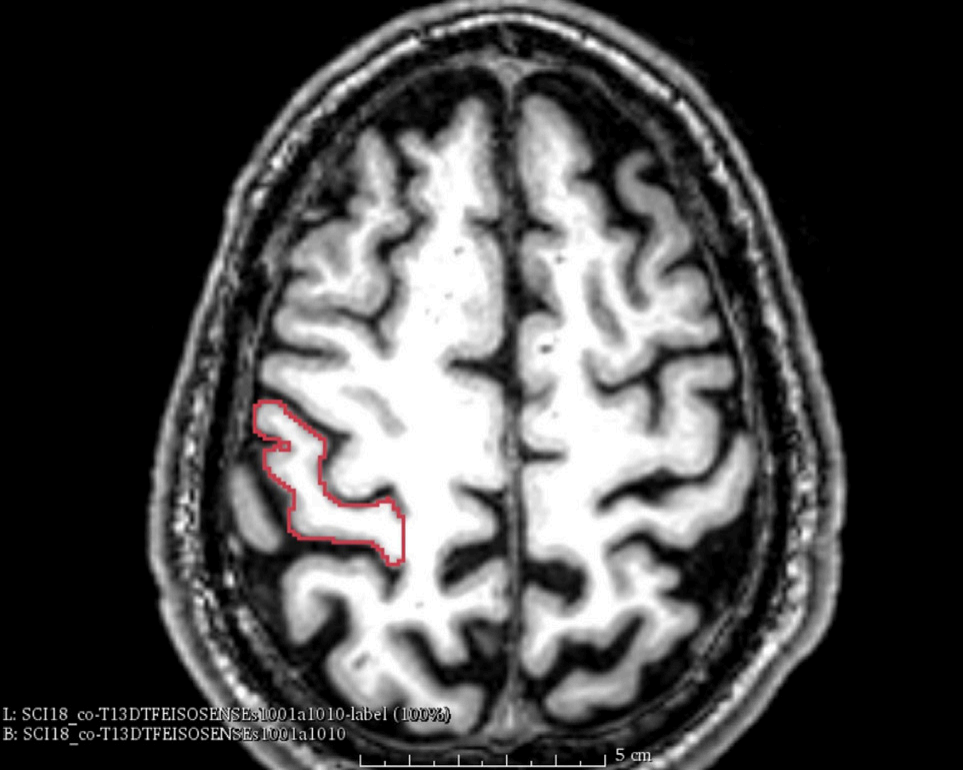
Omega sign. The omega sign in the central sulcus and the outlined somatosensory cortex (patient nr. 8).

**Figure 2:**
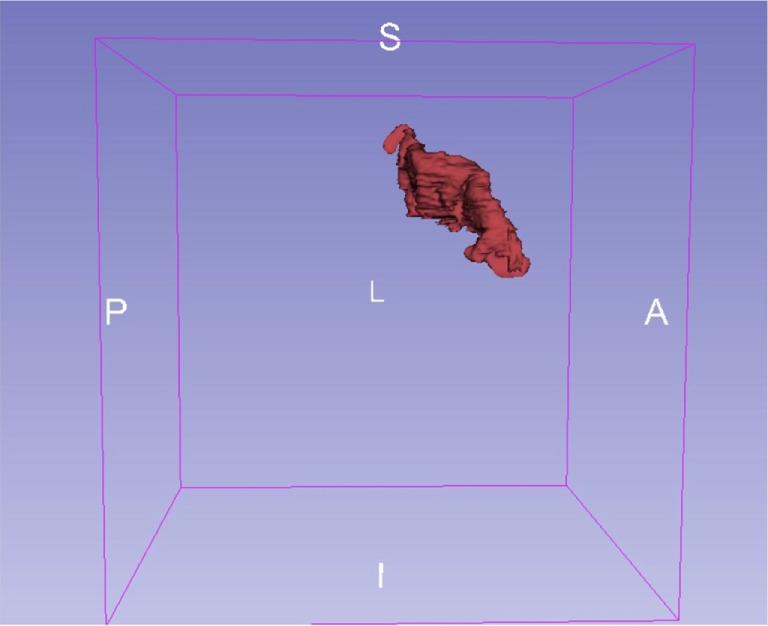
Segmentation. Exemplary 3D-model of the somatosensory cortex (patient nr. 8).

We used anatomical landmarks such as the *Omega sign*, as shown in Figure 1. The central sulcus separates the pre- and postcentral gyri, where the latter contains the primary somatosensory cortex (S1). It has three genua, of which the middle genu is shaped like an inverted greek letter Omega, which is seen in axial sections of MRIs. This structure is more colloquially called “hand-nob.” Identification of the central sulcus is essential in the topographic localization of the postcentral gyrus (33). The method used was to mark the outline of the somatosensory cortex in each individual plane in sagittal, coronal, axial order with permanent control in all planes. The volume of the segmented region was calculated and shown as a 3D-model (see Figure 2).

### 2.4 Normalization by Automated Segmentation

The automated segmentation was performed using a set of 30 hand labeled atlases (83 regions each) made publicly available by Hammers et al. (34).^1^ After brain extraction using BET in FSL,^2^ all subjects were diffeomorphically registered using ANTS^3^ to each atlas. The final segmentation is obtained by using majority voting to fuse the registration outcomes for each subject. The result is a labeled volume, containing labels for various different cortical and subcortical structures.

The volume of the manually segmented primary somatosensory cortex was normalized by the sum of the volumes of the following regions of the brain on both sides, which were obtained from the automated segmentation: temporal, occipital, and parietal lobe; temporal, frontal, parietal, fusiform, pre- and postcentral, straight, orbital, lingual, ambient, parahippocampal, and cingulate gyrus; and hippocampus, amygdala, caudate nucleus, putamen, nucleus accumbens, globus pallidus, substantia nigra, cerebellum, brainstem, insula, thalamus, corpus callosum, ventricles, cuneus, presubgenual and subgenual frontal cortex, and the subcallosal area. Normalization means that we calculated the percent the primary somatosensory cortex took in relation to the sum of the automatically segmented regions.

The sum of all these regions was on average 1,119,604.18 mm^3^ in healthy controls (SD = 122,183.51) and 1,041,145.67 mm^3^ in patients (SD = 111,013.47), which is a bit less than what would be expected for the total brain size in humans (35), seeming plausible since not all brain regions are included in this automated segmentation.

### 2.5 Statistical Analysis

Since the sample was quite small in comparison with the number of factors, we chose a non-parametric MANOVA for repeated measures designs. The R-package MANOVAM (36) allows to calculate test statistics in semi-parametric multivariate data with repeated measures designs. We used it with the parametric bootstrap and 1,000 iterations, because in contrast to the permutation or wild bootstrap, the parametric bootstrap is more robust in unbalanced designs and, therefore, generally recommended (37).

We performed three separate analyses. One analysis compared patients with controls in terms of normalized volume of the left- and right somatosensory cortex, taking also sex into account.

The patient group was divided into a homogeneous group of young patients with an age range of 22–26 years, and an older group aged 50 years and above. We could not include age into the statistical model applied to the comparison of patients with healthy controls since all healthy subjects were younger than the older patient group boarder age. For the sake of completeness, we compared patients and healthy controls in terms of age (*t*-test) and sex (Fisher’s exact test).

Therefore, two further analyses were performed in order to examine clinical effects in the patients group, thus, by taking into account lesion (cervical/thoracic), time since injury, age, and region. We could not put all factors in one single analysis since the subgroups were too small. Thus, in a second analysis, the subsamples of the patients were compared statistically by age, level, and region, and in a third analysis by duration, level and region. Similar to the division of the patient group into a younger and older group, we grouped patients with a duration below and above 84 months, since this divided the sample into two homogeneous subgroups. In addition, the sample of the related study of Freund et al. (5) included only patients with a duration of at least 84 months.

The results of the three separate MANOVAs were interpreted according to Bonferroni at *p* < 0.0167. For all test results, we report the Wald-Type-Statistic (WTS) alongside with degrees of freedom (df) and the p-value.

In order to report the distribution of the sample we plotted the means together with the confidence intervals as provided by the package MANOVAM.

## 3 Results

Clinical details of the included 11 patients are given in Table 1.

The healthy sample differed significantly from the patients with respect to age (2-sample *t*-test: age at onset: *t*(21) = –3.51; *p* = 0.0021; age at MRI: *t*(21) = –6.37; *p* = 0.000002) but not sex (Fisher’s exact test *OddsRatio* = 0.4082; *p* = 0.41).

**Figure 3:**
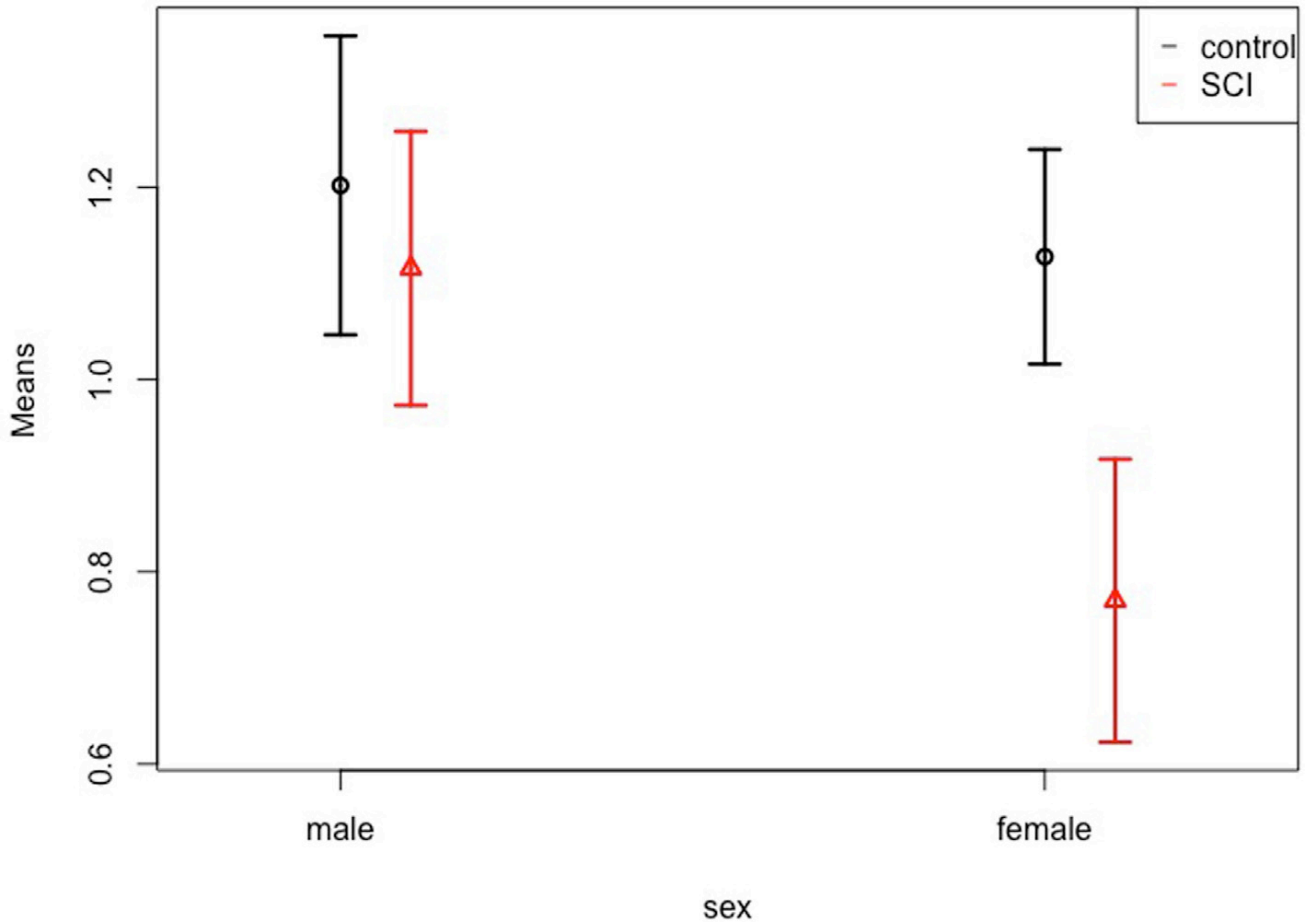
Volumes of patients vs. controls. Normalized volume means of the somatosensory cortex in controls and patients, separately for females and males. The whiskers represent the 95% confidence interval.

Patients showed smaller volumes than controls (*WTS* = 6.43; *df* = 1; *p* = 0.011), and female participants showed a tendency toward smaller volumes than males (*WTS* = 5.75; *df* = 1; *p* = 0.017). There was no effect of hemisphere (left vs. right), interaction of group and sex, group and region, sex and region, and of all three factors together. Figure 3 shows the means and confidence intervals of normalized volumes in patients and controls, separately for females and males. In addition to the Figure, Table 2 gives the exact numbers of the effects.

**Table 2:** Means and confidence intervals for the examined effects of the MANOVA for group (patients vs. controls), region (normalized volume of the left- and right somatosensory cortex), and sex.

Group	Sex	Region	n	Mean	Lower 95% CI	Upper 95% CI
Control	m	Left	5	1.192	0.512	1.872
Control	m	Right	5	1.212	0.762	1.662
Control	f	Left	7	1.063	0.794	1.331
Control	f	Right	7	1.193	0.850	1.536
SCI	m	Left	7	1.139	0.676	1.601
SCI	m	Right	7	1.093	0.726	1.460
SCI	f	Left	4	0.742	0.220	1.265
SCI	f	Right	4	0.798	0.057	1.538

*n, size of subgroup; m, male; f, female; CI, confidence interval*.

**Figure 4:**
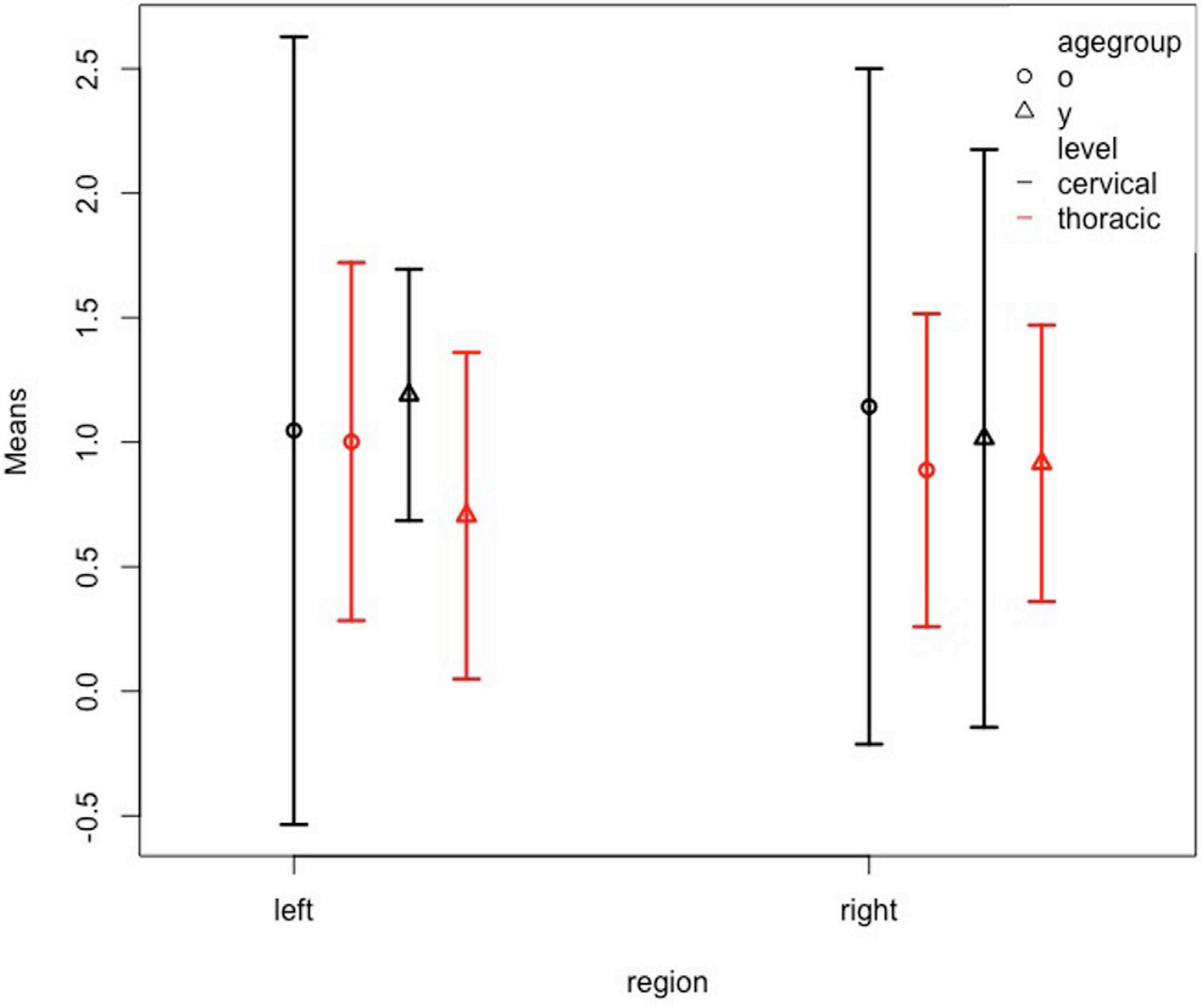
Left and right somatosensory cortex volumes of patient subgroups by age and level. Normalized volume means of the left and right somatosensory cortex for patients grouped by age group (y = ≤26 years; o = ≥50 years) and level (cervical; thoracic). The whiskers represent the 95% confidence interval.

Next, the analysis of patient subgroups by age and level, including also region (left vs. right hemisphere) revealed no main effects for age, level, or region, and no interactions despite a significant three-way interaction of all three factors age, level, and region (*WTS* = 14.48; *df* = 1; *p* < 0.001). Figure 4 shows the confidence intervals for this interaction. It must be considered that this three-way interaction is merely due to the small sample sizes since the confidence intervals largely overlap. Again, a table (Table 3) gives the exact numbers of the effects.

**Table 3:** Means and confidence intervals for the examined effects of the MANOVA for age (≥50 vs. ≤26 years), level (cervical vs. thoracic), and region (normalized volume of the left- and right somatosensory cortex).

Age (year)	Level	Region	n	Mean	Lower 95% CI	Upper 95% CI
≥50	Cervical	Left	3	1.047	−0.535	2.628
≥50	Cervical	Right	3	1.143	−0.213	2.500
≥50	Thoracic	Left	4	1.002	0.284	1.721
≥50	Thoracic	Right	4	0.888	0.259	1.516
≤26	Cervical	Left	2	1.190	0.685	1.695
≤26	Cervical	Right	2	1.015	−0.145	2.175
≤26	Thoracic	Left	2	0.705	0.049	1.361
≤26	Thoracic	Right	2	0.915	0.360	1.470

*n, size of subgroup; CI, confidence interval*.

**Figure 5:**
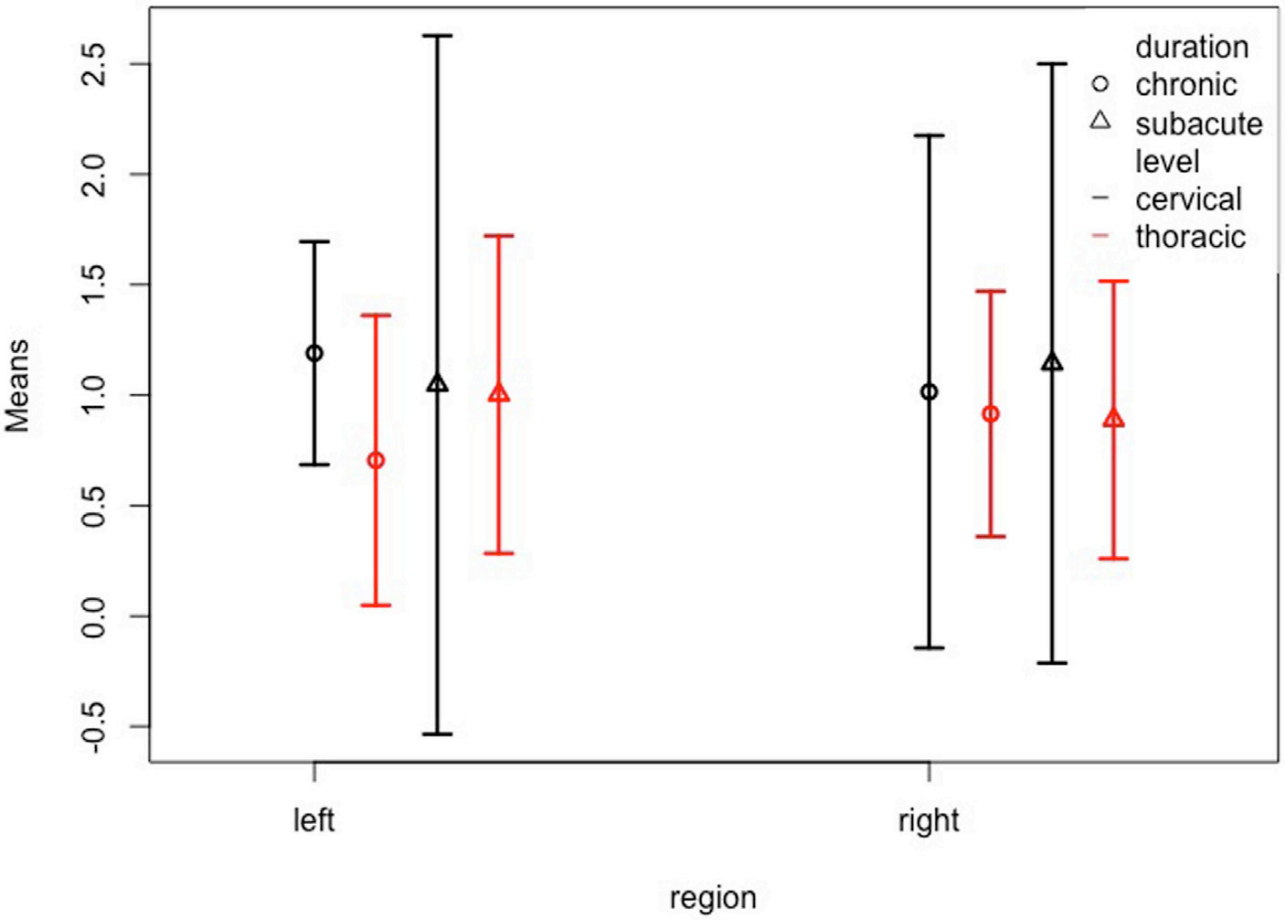
Left and right somatosensory cortex volumes of patient subgroups by duration and level. Normalized volume means of the left and right somatosensory cortex for patients grouped by duration (subacute = less than 84 months; chronic = more than 84 months) and level (cervical; thoracic). The whiskers represent the 95% confidence interval.

Finally, we examined the possible interaction between duration, level, and region, and again found no significant effects despite a three-way interaction of all factors duration, level, and region (*WTS* = 14.49; *df* = 1; *p* < 0.001). Figure 5 and Table 4 show the confidence intervals for this interaction. Again, the confidence intervals overlap largely, so that it must be considered that the effect is rather due to a statistical artifact. For example, for the chronic cases with durations >84 months, we should not be tempted to draw conclusions, since there were only 4 cases, two of which with a thoracic lesion and the other two with an unknown level of lesion.

**Table 4:** Means and confidence intervals for the examined effects of the MANOVA for duration (>12 vs. ≤12 months), level (cervical vs. thoracic), and region (normalized volume of the left- and right somatosensory cortex).

Duration (month)	Level	Region	n	Mean	Lower 95% CI	Upper 95% CI
>12	Cervical	Left	2	1.190	0.685	1.695
>12	Cervical	Right	2	1.015	−0.145	2.175
>12	Thoracic	Left	2	0.705	0.049	1.361
>12	Thoracic	Right	2	0.915	0.360	1.470
≤12	Cervical	Left	3	1.047	−0.535	2.628
≤12	Cervical	Right	3	1.143	−0.213	2.500
≤12	Thoracic	Left	4	1.002	0.284	1.721
≤12	Thoracic	Right	4	0.888	0.259	1.516

*n, size of subgroup; CI, confidence interval*.

## 4 Discussion

We found a lower volume of S1 in patients with incomplete SCI compared to controls, and a lower volume of the somatosensory cortex in females than in males, and also an interaction between the factors age, level of injury, and region, as well as duration, level of injury, and region. Our data suggest that our results are in line with previous findings (3, 5, 17, 18, 25–28), who reported smaller gray matter volume in the sensory or motor cortex of patients with SCI compared to controls.

The results might be of importance for neuroprosthetic devices, such as brain–spine interfaces that where recently developed in order to translate the signals from the central nervous system to the spinal cord in monkeys (38). However, the findings about atrophy of the central nervous system are also quite divergent with respect to the region and it is necessary to apply robust methods in order to clarify the true nature of the effects. Table 5 summarizes the findings of related studies.

**Table 5:** Summary of studies reporting gray matter volume changes in sensorimotor areas after SCI.

Study	f:m	Age	Time	c:t:ls	c:i	Method	Cortex	Effects
Crawley et al.^a^ (29)	4:13	33.1 ± 8.9	1–160	17:0:0	10:7	VBM ROI and manual	M1	No differences
Jurkiewicz et al.^a^ (25)	4:13	33.1 ± 8.9	1–160	17:0:0	10:7	VBM ROI	Bilateral S1	↓ vs. controls
Wrigley et al. (3)	0:15	41 ± 3	24–390	0:15:0	15:0	VBM global	M1	↓ vs. controls
Freund et al. (5)	0:10	47.1 ± 10.7	7–30	10:0:0	2:8	VBM global/ROI	M1	↓ vs. controls
							S1	↓ vs. controls
Henderson et al. (17)	2:18	38 ± 3	24–444	20:0:0	20:0	VBM ROI	Bilateral S1	↓ vs. controls
Freund et al. (18)	1:12	46.9 ± 20.2	1–12	8:5:0	4:9	VBM ROI	Left M1	↓ vs. controls
Hou et al. (26)	9:11	36.3 ± 5.6	2.5 ± 0.5	^b^	7:13	VBM ROI	M1	↓ vs. controls
							S1	↓ vs. controls
							M2	↓ vs. controls
Mole et al. (27)	^b^	52.5 ± 12.6	12–480	18:12:0	^b^	VBM global/ROI	Bilateral S1	↓ Patients with vs. without pain
							Bilateral S1	↑ patients without pain vs. controls
Villiger et al. (39)	4:5	55.1 ± 15.8	>12	5:4:0	0:9	VBCT ROI	Left M1	↓ vs. controls
						VBM/VBCT	Whole brain	No differences
Jutzeler et al. (28)	3:27	46.3 ± 11.9	24–324	15:13:2	11:19	VBM global/ROI	Left S2	↓ vs. controls
							Right M1	↑ Patients with vs. without pain
							Right S1	↓ Patients with vs. without pain
Chen et al. (30)	6:15	50.5 ± 12.1	1–396	8:1:12	10:11	VBM global	Only non-motor	↓ vs. controls

*All studies included also a sample of healthy controls; f:m, female to male ratio; time, time since injury in months*.

*^a^Crawley et al. (29) and Jurkiewicz et al. (25) analyzed the same sample*.

*^b^Indicate missing information*.

*c:t:ls, cervical:thoracic:lumbal or sacral; c:i, complete:incomplete; M1, primary motor; S1, primary somatosensory; M2, supplementary motor; S2, secondary somatosensory; VBM, voxel-based morphometry; global, global brain level; ROI, region of interest analysis*.

The consequence of deafferentation is apoptotic cell death in axotomized motorneurons in patients with SCI; accordingly, prevention of apoptosis could be established as a new target in therapeutic strategies (11). It is, therefore, of interest, which factors interact with neuronal loss and, thus, atrophy in the sensorimotor cortex.

### 4.1 Time Since Injury

The 10 patients in Freund et al.’s study (5) all had a duration since injury of at least 84 months, whereas in our sample all but four patients had a duration of up to 1 year. It is of interest that still, our data seem to replicate the lower volumes of patients compared to controls. However, our results with respect to an interaction with duration are rather unspecific and due to the small sample of patients with a longer duration this effect should not be overinterpreted.

In a latter, prospective study, Freund et al. reported volumetric loss in the left primary motor cortex (18). In this study, 13 patients were followed for 1 year, which is the time-span covered by our cross-sectional sample. It seems that volumetric changes take place early in time after SCI. Indeed, also in the study of Chen et al., no relation to duration could be established (30).

Increased cortical reactions seen in functional magnetic resonance imaging to electric stimulation of the front paws were detected as early as 7 days after partial cervical SCI in rats, indicating a functional connection between the cortical areas of forelimbs and hindlimbs (40). Most interestingly, in patients with a complete spinal cord injury at the C5 and C6 level, the spared contralateral biceps brachialis muscle projected at an enlarged cortical map within 6 days after injury (41).

Non-human primate studies revealed that fine motor skills after SCI are initially harmed, but have a high potential of improvement within a few weeks with practice, if the SCI is limited to the lateral corticospinal tract, that is the straight conjunction between cortical and motorneural structures, and if the SCI is at C4/C5 (42–44). This is of interest because the subregions of the sensorimotor cortex have distinctive involvement over the course of rehabilitation (43).

### 4.2 Age at Injury

Based on our results, we cannot assume that older patients are in general more susceptible to loss of volume of the somatosensory cortex after SCI. There was no difference in the volumes of the left or right somatosensory cortex in patients encountering injury at an age of 50 years and older vs. up to the age of 26. Thus, the documented volumetric and microstructural bilateral changes with age in the postcentral gyrus (45) might not interact with neuroplasticity after SCI. However, our results suggest that the interaction of several factors, namely the higher age and the more severe injury, i.e., at a cervical rather than at a thoracical injury, may lead to increased atrophy. Nevertheless, also in this interaction, we should be cautious since the size of the subgroups is rather small.

### 4.3 Incomplete Lesions

In contrast to previous studies, our sample involved exclusively incomplete SCI. It is remarkable that still, we could replicate previous findings of atrophy in S1. A recent study by Chen et al. (30) explored whole brain gray matter volumes and reported significant atrophy of patients with SCI compared to healthy controls only for the left anterior insular cortex, left and right orbitofrontal cortex, and right anterior insular cortex. These areas showed no difference between patients with complete and incomplete SCI. It is of interest that when the statistics are applied to the whole brain, a change in the sensorimotor region is not detectable. It is possible that this region’s atrophy is not dominant enough to become significant because other regions exhibit larger effects. We could hypothesize that this effect occurs in analogy to the effect of age, where other regions exhibit stronger atrophy than the postcentral gyrus (45).

The main symptom of patients with incomplete injury is sensory impairments. In such a sample, neuropathic pain is a frequent nuisance. Unfortunately, because of the retrospective nature of our study, we could not retrieve complete information on neuropathic pain in the examined sample. Jutzeler et al. (28) reported increased volumes for patients with neuropathic pain vs. patients without neuropathic pain in the right primary motor cortex. Mole et al. (27) reported reduced gray matter volume in the deafferented leg area of the bilateral somatosensory cortex when contrasting patients with vs. without neuropathic pain and increased volume of the bilateral primary somatosensory cortex in patients without pain compared to controls.

### 4.4 Sex

It is worth mentioning that specifically women with SCI showed a smaller volume of the somatosensory cortex than men with SCI. The effect was found in the overall sample and the interaction of group vs. sex was not significant. Nevertheless, the data suggest that the effect is mainly due to a difference between men and women in the patients group, while the difference is negligible in the healthy control group. It is possible that our sample was too small in order to identify this effect statistically.

Women have in general a higher volume in the cortical regions of the brain than men, but men have a higher volume in the subcortical regions of the brain (46). Relating this difference between women and men to our results could make us speculate that specific cortical regions are not only larger in women than in men but also more susceptible to loss of volume after SCI. However, since our normalization technique involved both cortical and subcortical regions, the difference is unlikely to be attributed to general sex differences.

### 4.5 Delineation of the Region of Interest

Our study applied a manual segmentation approach, which is surely more time-consuming than voxel-based morphometry that was used in all other studies; only one study implemented additional manual measurement of cortical thickness (29). The manual segmentation and normalization by sum of other brain regions, however, takes into account the size of the brain that varies with other factors such as age and sex and, thus, may serve to delineate the relative atrophy of the region of interest compared to other regions.

We reported recently that, with increasing atrophy, automated segmentation may fail to accurately delineate the shape of the region of interest (31, 32). However, manual segmentation of the whole brain is unfeasible, so that we relied on automated segmentation for normalization purposes. Another possible alternative could be to compare the somatosensory cortical volume to the total intracranial volume. It is possible that the normalization by total intracranial volume is more strongly interacting with age, since there is some global atrophy with age, which needs to be considered in normalization (47). However, a strong interaction with age would be undesirable within the present research context; it would overestimate the difference between controls and patients, since our control group was very young.

Voxel-based morphometry seems to be the *de facto* standard when looking at Table 5. However, the selection of the ROI is crucial when it comes to statistical significance. It was suggested that statistical weaknesses are the source of the so-called reproducibility crisis (48). In recent publications on cortical atrophy in SCI, corrections for multiple comparisons were, if at all, not applied to all tests, e.g., only to within-cluster tests, which needs to be considered when comparing the diverging results. For example, Chen et al. (30) performed a strict global analysis of voxel-based morphometry and found changes only in non-motor regions. The choice of the region of interest alongside with the statistical methods used is, thus, determining whether the small effect in the central motor cortex appears to be significant.

### 4.6 Limitations

There are several limitations of our study, which are due to the retrospective design. The MRIs were taken at highly variable time since injury (1–312 months). Thus, we could not compare each patients MRIs in determined timed intervals as in previous work (18). Moreover, part of the patient’s MRIs were performed with a different MR-sequence, at a different scanner. This is due to the situation at our institution, where we have two scanners: one for clinical purposes and one for research. The patients were partly extracted from the clinical database and, thus, were scanned at the clinical (Philips) facilities, while the controls and patients who participated in our study were scanned at the research MRI (Siemens). Due to the fact that the clinics and all other MRI institutes in our region work to capacity, it would not be feasible to examine controls at the clinical MRI facilities.

A further consequence of the retrospective nature of the study is missing data on additional factors that might better explain our findings. For example, the distinction between traumatic and non-traumatic injury might be of relevance, as well as the occurrence of neuropathic pain (27, 28), surgical interventions, or medication.

Another major problem is the young age of the controls, which was due to the fact that they were recruited among the students of our University. The normalization technique employed should be able to account for this; however, we cannot rule out that there was a bias of age.

A major weakness of all studies in this field, including ours, is the small sample size and, thus, power. In Table 5, 8 out of 11 studies are based on samples with *N* ≤ 20. Our sample is of 11 patients and Table 5 lists 2 out of 11 studies have sample sizes smaller than 12. However, in the study of Freund et al. (5), the sample consisted of a sample that was homogeneous such that all 10 patients had a cervical lesion, and in the sample of Villiger et al. (39), all 9 patients suffered from an incomplete lesion (AIS Grade D). Also our sample included only patients with an incomplete lesion (AIS Grade C–D), but still, there was an inhomogeneity in terms of age, sex, time since injury, and level of the lesion.

The small sample and especially the small size of the subsamples needs to be considered when interpreting the group interactions. Specifically, the three-way interactions may be rather a statistical artifact rather than a true effect. It would be very much expected that level of lesion may cause differential atrophic changes, so that we included this factor in our analysis. However, the results from the three-way interactions alongside with Figures 4 and 5 do only suggest that the variance is greater in the subgroup with a cervical lesion than in the subgroup with a thoracic lesion. The small sample sizes might also explain why other studies did not report on differences between subgroups, because multiple testing would have destroyed the power of the test. While we must be cautious with the interpretation of non-significant effects, it is highly advisable to set out for multicentric studies, international consortia, and meta-analyses in this field of research.

### 4.7 Conclusion and Future Directions

It was repeatedly claimed that SCI leads to atrophy of the sensorimotor cortex. Since we assume that axons, originating in the somatosensory cortex, play an important role in the functional recovery of patients with spinal cord injury, a better understanding of the neuroplastic changes, depending on the type and height of lesion, is stringently required for rehabilitative programs (16) and for the futuristic neuroprostheses that are directly interfacing with the central nervous system (23, 24, 38). However, since atrophy seems to occur after shortest periods of time, future rehabilitation programs must be implemented immediately. The establishment of therapies such as neuroprostheses should also consider that there might be additional factors that moderate neuroplastic changes, such as sex. These factors could interact with the functioning of futuristic neuroprosthetic devices, especially when such a device leads to a reversal of the atrophy caused by spinal cord injury.

As a result of reviewing the literature we suggest that multicenter studies or data pooling across research groups could shed further light on the factors contributing to central nervous atrophy after SCI, since the combination of samples would help to achieve the necessary statistical power.

## Ethics Statement

This study was carried out in accordance with the recommendations of Good Clinical Practice. From participants that were involved during a scientific study, we obtained written informed consent in accordance with the Declaration of Helsinki. The protocol was approved by the Ethics Commission Salzburg (Ethikkommission Land Salzburg; approval number 1541). From the other participants who were not involved in the scientific studies, we used clinical routine data retrospectively, extracted from the clinical database in anonymized form.

## Author Contributions

YH wrote the first draft of the paper and implemented all comments from the coauthors to create the final version, calculated the statistics, and prepared statistical figures. AT performed the segmentation of the cortices, contributed to the writing, and prepared the segmentation figures. ACT, SL, and PH acquired and gathered the data. CH calculated automated segmentation of brain volumes for normalization purposes. ST trained and supervised AT in manual segmentation of the somatosensory cortex. ET, SL, and RN supervised the work in clinical respects. All of the listed authors have read and commented during writing, and approved the final manuscript.

## Conflict of Interest Statement

The authors declare that the research was conducted in the absence of any commercial or financial relationships that could be construed as a potential conflict of interest.

## Footnotes

^1^Available from: http://brain-development.org/brain-atlases/individual-adult-brain-atlases-30/.

^2^Available from: http://fsl.fmrib.ox.ac.uk.

^3^Available from: http://stnava.github.io/ANTs/.

## References

[1] Dietz V Curt A. Neurological aspects of spinal-cord repair: promises and challenges. Lancet Neurol (2006) 5(8):688–94. 10.1016/S1474-4422(06)70522-1 16857574

[2] Jurkiewicz MT Mikulis DJ McIlroy WE Fehlings MG Verrier MC. Sensorimotor cortical plasticity during recovery following spinal cord injury: a longitudinal fMRI study. Neurorehabil Neural Repair (2007) 21(6):527–38. 10.1177/1545968307301872 17507643

[3] Wrigley PJ Gustin SM Macey PM Nash PG Gandevia SC Macefield VG Anatomical changes in human motor cortex and motor pathways following complete thoracic spinal cord injury. Cereb Cortex (2009) 19(1):224–32. 10.1093/cercor/bhn072 18483004

[4] Kokotilo KJ Eng JJ Curt A. Reorganization and preservation of motor control of the brain in spinal cord injury: a systematic review. J Neurotrauma (2009) 26(11):2113–26. 10.1089/neu.2008.0688 19604097 PMC3167869

[5] Freund P Weiskopf N Ward NS Hutton C Gall A Ciccarelli O Disability, atrophy and cortical reorganization following spinal cord injury. Brain (2011) 134(6):1610–22. 10.1093/brain/awr093 21586596 PMC3102242

[6] Lundell H Barthelemy D Skimminge A Dyrby TB Biering-Sørensen F Nielsen JB. Independent spinal cord atrophy measures correlate to motor and sensory deficits in individuals with spinal cord injury. Spinal Cord (2011) 49(1):70–5. 10.1038/sc.2010.87 20697420

[7] Freund P Curt A Friston K Thompson A Tracking changes following spinal cord injury: insights from neuroimaging. Neuroscientist (2013) 19:116–28. 10.1177/1073858412449192 22730072 PMC4107798

[8] Beaud M-L Schmidlin E Wannier T Freund P Bloch J Mir A Anti-Nogo-A antibody treatment does not prevent cell body shrinkage in the motor cortex in adult monkeys subjected to unilateral cervical cord lesion. BMC Neurosci (2008) 9(1):5 10.1186/1471-2202-9-5 18194520 PMC2242790

[9] Wall JT Xu J Wang X. Human brain plasticity: an emerging view of the multiple substrates and mechanisms that cause cortical changes and related sensory dysfunctions after injuries of sensory inputs from the body. Brain Res Brain Res Rev (2002) 39(2–3):181–215. 10.1016/S0165-0173(02)00192-3 12423766

[10] Dusart I Schwab ME Secondary cell death and the inflammatory reaction after dorsal hemisection of the rat spinal cord. Eur J Neurosci (1996) 6(5):712–24. 10.1111/j.1460-9568.1994.tb00983.x 8075816

[11] Hains BC Black JA Waxman SG. Primary cortical motor neurons undergo apoptosis after axotomizing spinal cord injury. J Comp Neurol (2003) 462(3):328–41. 10.1002/cne.10733 12794736

[12] Green JB Sora E Bialy Y Ricamato A Thatcher RW. Cortical sensorimotor reorganization after spinal cord injury: an electroencephalographic study. Neurology (1998) 50(4):1115–21. 10.1212/WNL.50.4.1115 9566404

[13] Green JB Sora E Bialy Y Ricamato A Thatcher RW. Cortical motor reorganization after paraplegia: an EEG study. Neurology (1999) 53(4):736–43. 10.1212/WNL.53.4.736 10489034

[14] Aguilar J Humanes-Valera D Alonso-Calviño E Yague JG Moxon KA Oliviero A Spinal cord injury immediately changes the state of the brain. J Neurosci (2010) 30(22):7528–37. 10.1523/JNEUROSCI.0379-10.2010 20519527 PMC3842476

[15] Cohen-Adad J El Mendili MM Lehéricy S Pradat PF Blancho S Rossignol S Demyelination and degeneration in the injured human spinal cord detected with diffusion and magnetization transfer MRI. Neuroimage (2011) 55(3):1024–33. 10.1016/j.neuroimage.2010.11.089 21232610

[16] Ellaway PH Kuppuswamy A Balasubramaniam AV Maksimovic R Gall A Craggs MD Development of quantitative and sensitive assessments of physiological and functional outcome during recovery from spinal cord injury: a clinical initiative. Brain Res Bull (2011) 84(4–5):343–57. 10.1016/j.brainresbull.2010.08.007 20728509

[17] Henderson L Gustin S Macey P Wrigley P Siddall P. Functional reorganization of the brain in humans following spinal cord injury: evidence for underlying changes in cortical anatomy. J Neurosci (2011) 31:2630–7. 10.1523/JNEUROSCI.2717-10.2011 21325531 PMC6623700

[18] Freund P Weiskopf N Ashburner J Wolf K Sutter R Altmann D MRI investigation of the sensorimotor cortex and the corticospinal tract after acute spinal cord injury: a prospective longitudinal study. Lancet Neurol (2013) 12:873–81. 10.1016/S1474-4422(13)70146-7 23827394 PMC3744750

[19] Wall PD Egger MD Formation of new connexions in adult rat brains after partial deafferentation. Nature (1971) 232(5312):542–5. 10.1038/232542a0 4328622

[20] Nardone R Höller Y Brigo F Seidl M Christova M Bergmann J Functional brain reorganization after spinal cord injury: systematic review of animal and human studies. Brain Res (2013) 1504:58–73. 10.1016/j.brainres.2012.12.034 23396112

[21] Flor H Elbert T Knecht S Wienbruch C Pantev C Birbaumer N Phantom-limb pain as a perceptual correlate of cortical reorganization following arm amputation. Nature (1995) 375(6531):482–4. 10.1038/375482a0 7777055

[22] Moore CI Stern CE Dunbar C Kostyk SK Gehi A Corkin S. Referred phantom sensations and cortical reorganization after spinal cord injury in humans. Proc Natl Acad Sci U S A (2000) 97(26):14703–8. 10.1073/pnas.250348997 11114177 PMC18982

[23] Rupp R Gerner HJ Neuroprosthetics of the upper extremity – Clinical application in spinal cord injury and challenges for the future. In: Sakas DE Simpson BA Krames ES editors. Operative Neuromodulation – Volume I: Functional Neuroprosthetic Surgery. An Introduction, Acta Neurochirurgica Supplement 97/1. New York: Springer-Verlag Wien (2007). p. 419–426 10.1007/978-3-211-33079-1_55 17691405

[24] Moxon K Foffani G. Brain-machine interfaces beyond neuroprosthetics. Neuron (2015) 86:55–67. 10.1016/j.neuron.2015.03.036 25856486

[25] Jurkiewicz M Crawley A Verrier M Fehlings M Mikulis D. Somatosensory cortical atrophy after spinal cord injury: a voxel-based morphometry study. Neurology (2006) 66:762–4. 10.1212/01.wnl.0000201276.28141.40 16534122

[26] Hou J Yan R Xiang Z Zhang H Liu J Wu Y Brain sensorimotor system atrophy during the early stage of spinal cord injury in humans. Neuroscience (2014) 266:208–15. 10.1016/j.neuroscience.2014.02.013 24561217

[27] Mole T MacIver K Sluming V Ridgway G Nurmikko T. Specific brain morphometric changes in spinal cord injury with and without neuropathic pain. Neuroimage Clin (2014) 5:28–35. 10.1016/j.nicl.2014.05.014 24936434 PMC4055864

[28] Jutzeler CR Huber E Callaghan MF Luechinger R Curt A Kramer JL Association of pain and CNS structural changes after spinal cord injury. Sci Rep (2016) 6:18534. 10.1038/srep18534 26732942 PMC4702091

[29] Crawley A Jurkiewicz M Yim A Heyn S Verrier M Fehlings M Absence of localized grey matter volume changes in the motor cortex following spinal cord injury. Brain Res (2004) 1028:19–25. 10.1016/j.brainres.2004.08.060 15518637

[30] Chen Q Zheng W Chen X Wan L Qin W Qi Z Brain gray matter atrophy after spinal cord injury: a voxel-based morphometry study. Front Hum Neurosci (2017) 11:211. 10.3389/fnhum.2017.00211 28503142 PMC5408078

[31] Liedlgruber M Butz K Höller Y Kuchukhidze G Taylor A Tomasi O Variability issues in automated hippocampal segmentation: a study on out-of-the-box software and multi-rater ground truth. Proceedings of the 29th IEEE International Symposium on Computer-Based Medical Systems. Dublin (2016). p. 191–6.

[32] Liedlgruber M Butz K Höller Y Kuchukhidze G Taylor A Thomschewski A Pathology-related automated hippocampus segmentation accuracy. Proceedings of Bildverarbeitung für die Medizin. Heidelberg: Springer Informatik Aktuell (2017). p. 128–33.

[33] Campero A Martins C de Alecantro L Ajler P Emmerich J Rhoton A. Usefulness of the contralateral omega sign for the topographic location of lesions in and around the central sulcus. Surg Neurol Int (2011) 2(1):164. 10.4103/2152-7806.89892 22140649 PMC3228394

[34] Hammers A Allom R Koepp M Free S Myers R Lemieux L Three-dimensional maximum probability atlas of the human brain, with particular reference to the temporal lobe. Hum Brain Mapp (2003) 19:224–47. 10.1002/hbm.10123 12874777 PMC6871794

[35] Allen JS Damasio H Grabowski TJ. Normal neuroanatomical variation in the human brain: an MRI-volumetric study. Am J Phys Anthropol (2002) 118:341–58. 10.1002/ajpa.10092 12124914

[36] Friedrich S Konietschke F Pauly M MANOVA.RM: A Package for Calculating Test Statistics and Their Resampling Versions for Heteroscedastic Semi-Parametric Multivariate Data or Repeated Measures Designs. R-Package Version 0.1.1. (2017). Available from: https://cran.r-project.org/web/packages/MANOVA.RM/index.html

[37] Bathke A Friedrich S Konietschke F Pauly M Staffen W Strobl N Using EEG, SPECT, and Multivariate Resampling Methods to Differentiate between Alzheimer’s and Other Cognitive Impairments. arXiv preprint arXiv:1606.09004 (2016).

[38] Capogrosso M Milekovic T Borton D Wagner F Moraud EM Mignardot J-B A brain–spine interface alleviating gait deficits after spinal cord injury in primates. Nature (2016) 539:284–8. 10.1038/nature20118 27830790 PMC5108412

[39] Villiger M Grabher P Hepp-Reymond M-C Kiper D Curt A Bolliger M Relationship between structural brainstem and brain plasticity and lower-limb training in spinal cord injury: a longitudinal pilot study. Front Hum Neurosci (2015) 9:254. 10.3389/fnhum.2015.00254 25999842 PMC4420931

[40] Ghosh A Sydekum E Haiss F Peduzzi S Zörner B Schneider R Functional and anatomical reorganization of the sensory-motor cortex after incomplete spinal cord injury in adult rats. J Neurosci (2009) 29(39):12210–9. 10.1523/JNEUROSCI.1828-09.2009 19793979 PMC6666156

[41] Streletz LJ Belevich JKS Jones SM Bhushan A Shah SH Herbison GJ. Transcranial magnetic stimulation: cortical motor maps in acute spinal cord injury. Brain Topogr (1995) 7(3):245–50. 10.1007/BF01202383 7599023

[42] Sasaki S Isa T Pettersson L-G Alstermark B Naito K Yoshimura K Dexterous finger movements in primate without monosynaptic corticomotoneuronal excitation. J Neurophysiol (2004) 92:3142–7. 10.1152/jn.00342.2004 15175371

[43] Nishimura Y Onoe H Morichika Y Perfiliev S Tsukada H Isa T. Time-dependent central compensatory mechanisms of finger dexterity after spinal cord injury. Science (2007) 318(5853):1150–5. 10.1126/science.1147243 18006750

[44] Alstermark B Pettersson LG Nishimura Y Yoshino-Saito K Tsuboi F Takahashi M Motor command for precision grip in the macaque monkey can be mediated by spinal interneurons. J Neurophysiol (2011) 106(1):122–6. 10.1152/jn.00089.2011 21511706

[45] Callaghan M Freund P Draganski B Anderson E Cappelletti M Chowdhury R Widespread age-related differences in the human brain microstructure revealed by quantitative magnetic resonance imaging. Neurobiol Aging (2014) 35:1862–72. 10.1016/j.neurobiolaging.2014.02.008 24656835 PMC4024196

[46] Ritchie SJ Cox SR Shen X Lombardo MV Reus LM Alloza C Sex differences in the adult human brain: evidence from 5,216 UK Biobank participants. bioRxiv (2017). 10.1101/123729 PMC6041980 29771288

[47] Peelle JE Cusack R Henson RNA. Adjusting for global effects in voxel-based morphometry: gray matter decline in normal aging. Neuroimage (2012) 60:1503–16. 10.1016/j.neuroimage.2011.12.086 22261375 PMC3898996

[48] Baker M 1,500 scientists lift the lid on reproducibility. Nature (2016) 533(7604):452–4. 10.1038/533452a 27225100

